# Hyperhomocysteinemia in a Patient with Moyamoya Disease

**DOI:** 10.1155/2018/7806873

**Published:** 2018-05-06

**Authors:** Durga Shankar Meena, Gopal Krishana Bohra, Mahadev Meena, Bharat Kumar Maheshwari

**Affiliations:** Department of Medicine, All India Institute of Medical Sciences, Jodhpur, Rajasthan, India

## Abstract

Moyamoya disease is a chronic progressive cerebrovascular disease characterized by bilateral occlusion or stenosis of arteries around circle of Willis. We report a case of 18-year-old female presented with recurrent episodes of headache and vertigo. On cerebral angiography, the patient was diagnosed to have moyamoya disease. On further evaluation, thrombophilia profile showed increased homocysteine level. The patient was treated conservatively with cobalamin and aspirin and advised for revascularization. According to the literature, there are few case reports of moyamoya disease with thrombotic disorders. Hence, we are reporting this interesting and rare case.

## 1. Introduction

Moyamoya disease is a poorly understood cerebrovascular disease with the reported incidence of 0.35 per 100,000 population [1]. Moyamoya in Japanese means “puff of smoke” which was used to describe the typical smoky angiographic appearance of collateral vessels. The etiology and pathogenesis of moyamoya disease are not well known. Familial occurrence is seen in approximately 10 percent of cases which suggests genetic etiology. There are few reports describing the classical angiographic appearance of moyamoya vessels with abnormal thrombophilia profile. Here, we present a case of 18-year-old female with moyamoya disease with hyperhomocysteinemia.

## 2. Case Presentation

**Figure 1:**
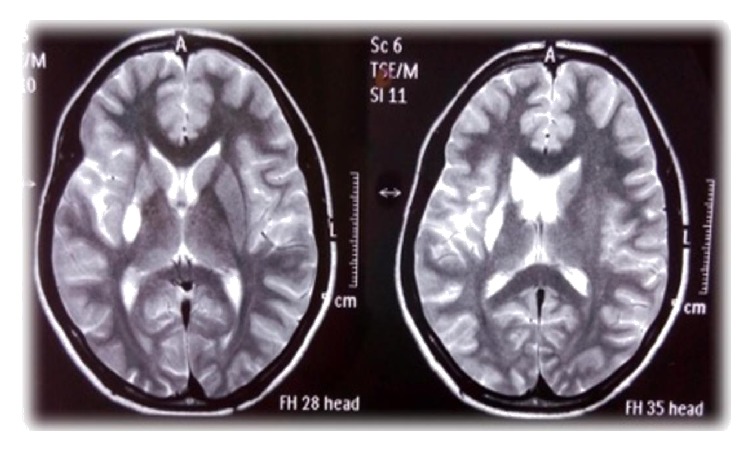
Axial T2-weighted MRI brain image showing infarct in right gangliocapsular region.

**Figure 2:**
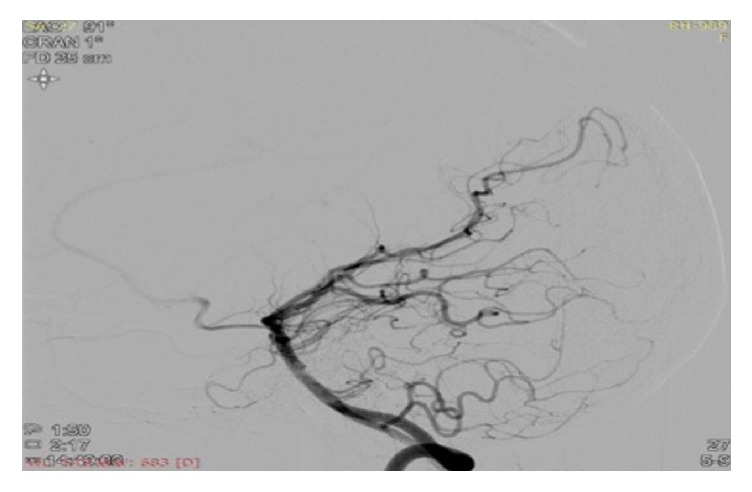
Vertebral angiogram image showing near total to complete occlusion of right M1 (MCA) just distal to origin with distal M1 reforming via perforators.

**Figure 3:**
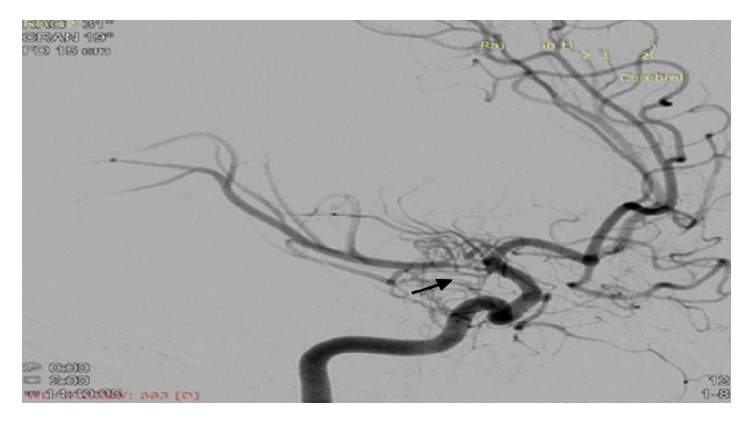
Cerebral angiogram (oblique view) image showing typical “puff of smoke” collaterals with right MCA stenosis (black arrow).

**Figure 4:**
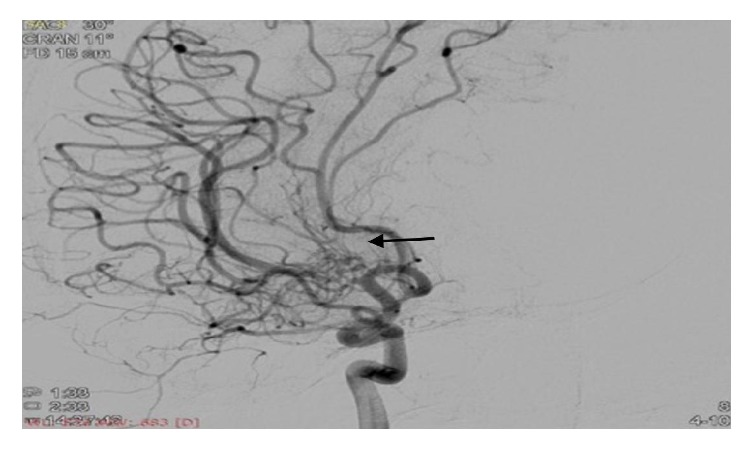
Cerebral angiogram image showing typical “puff of smoke” moyamoya collaterals (black arrow).

An 18-year-old female was admitted to our hospital with chief complaints of acute onset vertigo and severe headache for the last 6 hours. Soon after hospitalization, she developed 2 episodes of generalized tonic-clonic seizures. There was past history of left hemiparesis 8 years back with recurrent episodes of vertigo and headache. There was no history of fever, head injury, ear discharge, and diplopia. There was no significant family history. On general examination, patient's blood pressure was 150/78 mmHg at the time of admission, and rest examination was unremarkable. Her sensorium was improved after 1 hour. Higher mental functions were normal. The functions of cranial nerves were intact. Motor, sensory examination was normal and deep tendon reflexes were well preserved. The patient was further evaluated with biochemical and haematological investigations. Complete blood count, electrolytes, and blood sugar level were normal. MRI brain was done which showed infarct in right gangliocapsular region (Figure 1). We further evaluated the patient with cerebral angiography which showed occlusion of the right middle cerebral artery (M1) just distal to origin with the appearance of moyamoya collaterals (Figures 2, 3, and 4). Right internal carotid artery showed reduced calibre in comparison to left, likely due to stenosis. Since our patient has a history of stroke, we further investigated the underlying cause. On subsequent investigation for thrombophilia profile, homocysteine level was moderately increased (42 micromole/litre) (Table 1), and rest coagulation profile was unremarkable. We treated the patient conservatively with cobalamin, B-6, folic acid, and aspirin. The patient was advised for neurosurgery consultation for revascularization and further follow-up.

The patient successfully underwent surgical revascularization and at 3-month follow-up, the patient was symptomatically better.

## 3. Discussion

Moyamoya disease (MMD) is an uncommon chronic cerebrovascular disease characterized by the formation of collaterals near circle of Willis secondary to occlusion or stenosis of proximal, middle, or anterior cerebral artery. Moyamoya in Japanese means “puff of smoke” [2]. The etiology of moyamoya disease is unknown. 10% to 15% cases, especially in Japanese population, were reported to be familial. Patients with typical angiographic appearance without any known risk factor were considered moyamoya disease, while those with some recognized conditions (neurofibromatosis type 1, Down syndrome, cranial irradiation, and sickle cell disease) are classified as moyamoya syndrome. Abnormal thrombophilia profile in moyamoya disease is reported in the literature. There have been reports of moyamoya disease with protein c and protein s deficiency [3, 4]. One case report of homocystinuria with moyamoya was also described in the literature [5]. In our case, there was no clinical feature suggestive of homocystinuria. Cerrato et al. described a case of atherosclerotic moyamoya disease presented with ischemic stroke [6]. According to their report, atherosclerosis secondary to increased homocysteine was the likely cause of moyamoya.

Homocysteinemia mediated endothelial dysfunction, increased oxidant stress, and functional abnormalities in the release of nitric oxide are the proposed mechanism in some studies [7], leading to cerebrovascular disease. In our case, increase homocysteine could be an important trigger for the development of moyamoya vessels and stroke. The possible role of MTFHR (methylenetetrahydrofolate reductase) enzyme activity in MMD with hyperhomocysteinemia was discussed in the literature [8]. A recent study showed an association of 2 novel SNPs (single-nucleotide polymorphisms) in the gene regulating homocysteine metabolism (rs9651118 in MTHFR and rs117353193 in TCN2), causing increased homocysteine level in MMD patients [9]. However, due to financial constraints, we were not able to investigate for MTFHR gene mutation in our patient.

There is no curative treatment for moyamoya disease. Acute management is predominantly supportive with emphasis on reducing intracerebral pressure and management of seizures [10]. Secondary prevention for symptomatic moyamoya is predominantly based on surgical revascularization [11]. The goal of revascularization is to improve cerebral perfusion and prevent further risk of ischemic stroke. It is also important to look for underlying conditions such as coagulation profile.

In conclusion, our case, along with previous reports outlined above, recapitulates that homocysteinemia can be associated with moyamoya disease. The possibility of hyperhomocysteinemia should be sought in every patient presenting with moyamoya disease.

## Figures and Tables

**Table 1:** Biochemical and haematological investigations.

Investigations	
Homocysteine	42 *μ*mol/L (1.0–15.39)
ANA	Negative
Anti-Ro/SSA, anti-La/SSB	Negative
Protein C	78% (normal 70–120)
Protein S	134% (normal 55–135)
Antithrombin III	110% (normal 80–120)
Lupus anticoagulant	Absent
PT	12.1 seconds (control—11.1)
APTT	28.0 seconds (control—28.8)
LDL cholesterol	60.66 mg/dl
HDL cholesterol	40.08 mg/dl
Triglycerides	122.49 mg/dl
Cholesterol	155 mg/dl
Vitamin B-12	223 pg/ml (normal 211–700)
Folate level	3.4 ng/ml (normal 2–20)
Total protein/albumin	7.0/4.0 gm/dl
Blood urea nitrogen/creatinine	33/0.94 mg/dl
Thyroid function test	T3—0.4 ng /dl FreeT4—0.9 ng/dl TSH—2.9 mIU/L
